# Identification of Potential Biomarkers for Cancer Cachexia and Anti-Fn14 Therapy

**DOI:** 10.3390/cancers14225533

**Published:** 2022-11-10

**Authors:** Zhipeng Cao, Ingrid J. Burvenich, Kening Zhao, Clare Senko, Jason Glab, Renee Fogliaro, Zhanqi Liu, Irvin Jose, Hamsa Puthalakath, Nick J. Hoogenraad, Laura D. Osellame, Andrew M. Scott

**Affiliations:** 1 Department of Biochemistry and Genetics, La Trobe University, Melbourne, VIC 3086, Australia; 2 Tumour Targeting Laboratory, Olivia Newton-John Cancer Research Institute, School of Cancer Medicine, La Trobe University, Melbourne, VIC 3084, Australia; 3 Department of Laboratory Medicine, Nanfang Hospital, Southern Medical University, Guangzhou 510515, China; 4 Department of Molecular Imaging and Therapy, Austin Health, Heidelberg, VIC 3084, Australia; 5 Department of Medicine, University of Melbourne, Melbourne, VIC 3010, Australia

**Keywords:** cancer cachexia, biomarker, RNA-seq, LCN2

## Abstract

**Simple Summary:**

Cancer cachexia is an underestimated condition with huge impact on survival and quality of life for many cancer patients. Currently, there is no reliable diagnosis for this condition, particularly for cancer cachexia at an early stage. The aim of our study is to develop potential biomarkers that can be used not only for diagnosing cancer cachexia but also evaluating potential anti-cachexia therapy such as anti-Fn14 mAb treatment. We identified several circulating biomarkers for cancer cachexia and confirmed that LCN2 is a promising biomarker for cancer cachexia in humans. These biomarkers could accelerate both diagnosis and clinical trials for cancer cachexia.

**Abstract:**

Background: Developing therapies for cancer cachexia has not been successful to date, in part due to the challenges of achieving robust quantitative measures as a readout of patient treatment. Hence, identifying biomarkers to assess the outcomes of treatments for cancer cachexia is of great interest and important for accelerating future clinical trials. Methods: We established a novel xenograft model for cancer cachexia with a cachectic human PC3* cell line, which was responsive to anti-Fn14 mAb treatment. Using RNA-seq and secretomic analysis, genes differentially expressed in cachectic and non-cachectic tumors were identified and validated by digital droplet PCR (ddPCR). Correlation analysis was performed to investigate their impact on survival in cancer patients. Results: A total of 46 genes were highly expressed in cachectic PC3* tumors, which were downregulated by anti-Fn14 mAb treatment. High expression of the top 10 candidates was correlated with low survival and high cachexia risk in different cancer types. Elevated levels of LCN2 were observed in serum samples from cachectic patients compared with non-cachectic cancer patients. Conclusion: The top 10 candidates identified in this study are candidates as potential biomarkers for cancer cachexia. The diagnostic value of LCN2 in detecting cancer cachexia is confirmed in patient samples.

## 1. Introduction

Cancer cachexia is a multi-symptomatic disease, mainly characterized by muscle wasting, with or without loss of adipose tissue. Cachexia presents widely in many cancer types and is responsible for more than 20% of cancer related deaths [1]. Although the importance of cancer cachexia is well recognized, it has drawn little focus from both pharmaceutical companies and clinicians in the past. Cachexia and anorexia often present simultaneously, however, it has been shown that simply increasing food intake is usually ineffective in preventing or reducing weight loss in cachectic patients [2]. Recently, research has led to the development of various potential therapies to treat cancer cachexia, with a number reaching Phase III clinic trials [3].

As in other clinical trials, studies for cancer cachexia require reliable measurements to evaluate the efficacy of a treatment. Current measurements for assessing cancer cachexia include changes in body weight, a handgrip strength test, six-minute walk test and evidence of improvement of the quality of life which can be subjective and less significant in a short period of time [4]. Therefore, developing a sensitive method to objectively monitor and quantify the outcome of specific treatments for cancer cachexia is urgently required. Body composition measurement by Dual Energy X-ray Absorptiometry (DEXA) has also been used to assess cancer cachexia. The drawback is that the DEXA measurements cannot distinguish between other conditions that often occur with advanced cancer such as sarcopenia and anorexia. It is important to find ways of distinguishing between these groups of patients, and failure to do that could be one of the reasons for the disappointments of previous clinical trials for cancer cachexia. In addition, formal measurements of body composition cannot practically be performed in all cancer patients, and monitoring response to potential therapies would be ideally performed with a simple blood test.

Biomarker-based detection methods have been applied in many conditions, providing reliability and sensitivity in detecting these conditions. Some cachexia-inducing factors such as IL-6 were shown to be upregulated in preclinical cancer cachexia models, although none are widely increased in cachectic patients with different cancers [5,6]. Thus, establishing a reliable biomarker-based assay for cancer cachexia is not only important for diagnosis but would also be beneficial for monitoring outcomes of clinical studies of cancer cachexia.

Tumoral Fn14 has been shown to induce cancer cachexia in several preclinical models, with an antagonistic anti-Fn14 antibody (002 mAb) treatment able to reverse this condition [7], making Fn14 a potential target for treating cancer cachexia. It was also shown that the signal for cachexia originated in the tumour as Fn14-/- mice developed cachexia, dependent on tumoral Fn14 [7]. The current study was performed to identify potential biomarkers that can be used to diagnose cancer cachexia and monitor therapeutic treatments for this condition.

## 2. Materials and Methods

### 2.1. Cell Lines and Culture Conditions

PC3 (CRL-1435, ATCC) cells and the subclone that is referred to as PC3* cells, were cultured in RPMI 1640 (Invitrogen, Waltham, MA, USA) containing 10% fetal calf serum (FCS) and 1% penicillin/streptomycin at 37 °C with 5% CO_2_. Hras MEF (Mouse Embryonic Fibroblast) and Hras Fn14 MEF were cultured in high glucose DMEM (Invitrogen) containing 10% FCS and 1% penicillin/streptomycin at 37 °C with 5% CO_2_.

### 2.2. Development of a Human Cancer Cell Line Inducing Cachexia

The PC3 cell line is a widely used prostate cancer cell line, but we noticed morphological diversity in PC3 cells from ATCC. We discovered large differences in mass spectrometry clustering of single clones isolated from PC3. PC3* is a one of these clones which showed high homogeneity in morphology and principal component analysis. Short Tandem Repeat (STR) profiling was performed for cell line authentication, and we found that PC3 and PC3* cells had similar STR profiling, confirming that PC3* cells were derived from the parental PC3 cells. These two cell lines were inoculated in NSG mice to explore their differences in vivo, leading to our discovery that PC3* cells caused rapid weight loss compared with PC3 cells.

### 2.3. Animal Experiments

The Hras Fn14 MEF cancer cachexia model was established as previously reported [7]. Hras MEF (non-cachectic) or Hras Fn14 MEF (cachectic) cells were subcutaneously injected into C57BL/6 mice with 5 million cells per mouse. Mouse body weights were measured daily. Mice bearing Hras Fn14 MEF tumors were treated with 002 mAb at 10 mg/kg by intraperitoneal injection at day 7. Control mice were injected with the same volume of PBS. For the PC3* cancer cachexia model, 5 million PC3 or PC3* cells were subcutaneously injected into NOD scid gamma (NSG) mice. Mice received mAb 002 therapy twice per week via intraperitoneal injections (10 mg/kg) or PBS control from day 21 to the endpoint. At the endpoint, tumors were removed for transcriptomic analyses. All procedures in this study were performed under the institutes’ AEC approval. Plasma samples were collected by cardiac puncture into Microvette CB 300 K2E tubes (Sarstedt) followed by centrifugation at 2000× *g* for 5 min.

### 2.4. Cell Proliferation Assay

Cells were seeded in a 96-well plate with a density of 4000 cells per well. PBS control or 002 mAb (5 µg/mL) was added to the wells after the cells attached. To assess cell proliferation, 40 µL of MTS/PMS mixture (Promega, Madison, WI, USA) was added to each well with 200 µL culture medium. The plate was then incubated at 37 °C for 1 h, and absorbance at 490 nm was recorded using a plate reader.

### 2.5. Assessment of Muscle Cross-Sectional Area

Quadriceps muscle sections from animal experiments were fixed in formalin and embedded in paraffin wax. Muscles were sectioned using a microtome, placed on Superfrost slides (Thermo Fisher Scientific, Waltham, MA, USA) and dried for 1 h at 60 °C. Slides were then washed in xylene (2 × 5 min), 100% ethanol (2 × 3 min), 90% ethanol (3 min), 70% ethanol (3 min), water (1 min). Slides were stained in Hematoxylin (1 min), followed by washing with water (1 min), Scott’s solution (1 min) and water (1 min). Slides were stained with Eosin for 1 min, followed by washing in 100% ethanol (2 × 2 min) and xylene (2 × min). Slides were mounted using DFX Mountant (Sigma-Aldrich, St. Louis, MO, USA). Slides were scanned on an Aperio AT2 Slide Imager (Leica, Wetzlar, Germany). Each fiber was measured to obtain the cross-sectional area (CSA) of the muscle by ImageJ. Approximately 100 muscle fibers were traced per sample.

### 2.6. RNA Sequencing and Differential Gene Expression (DEG) Analysis

Total RNA was extracted using a RNeasy isolation kit (Qiagen) from 10 mg of PC3 (mouse number *n* = 3), PC3*(*n* = 6) and PC3* + 002 mAb (*n* = 5) tumors at the endpoint defined by vehicle control tumors reaching a size of 1000 mm^3^. The quality of the extracted RNA was assessed by 1% agarose gel. 2 µg of the extracted RNA was used to generate mRNA libraries using the TrueSeq Standard mRNA library kit (Illumina). Sequencing was performed using the NextSeq high output kit (75 cycles) (Illumina) on the Illumina NextSeq 500/550 platform (La Trobe University Genomics Platform). A total of 34,699,743 sequences were detected with sequence lengths of 34–76 bp. Low quality reads, adaptor reads and contaminants were removed from the raw sequencing using the trimgalore program (v 0.4.5). Resulting clean reads were aligned to the human genome (ENSEMBL GRch38) by HISat2 (v 2.1.0). RSeqQC (v2.6.4) was used to perform quality control of alignments. Gene expression was quantified as read counts by subread-feature counts (v.1.46p5) using ENSEMBL GRch38 genome annotation. EdgeR (v 3.18.1) was used for differential expression analysis and differentially expressed genes (DEGs) were identified using the following cut off thresholds: (RPKM > 5, FDR < 0.05 and log2FC > 1. Only genes meeting these criteria were considered significant. Clustering between samples and group comparison was analyzed using principal component analysis and visualized using multidimension scaling (RStudio). Gene products that were upregulated in PC3* (in comparison to PC3) and downregulated in Fn14-antibody treated tumors were subjected to *in silico* secretomic analysis using VerSeDa software [8]. Proteins encoded by these genes were manually curated and ranked using EXPASY (SignalP, SecretomeP, TMpred and TMHMM), UNIPROT and DeepLoc software.

### 2.7. ddPCR

Total RNA was extracted from tumors using TRIzol (Life Technologies, Carlsbad, CA, USA). RNA was dissolved in DEPC treated water and quantified using Nanodrop. Extracted RNA (2 µg) was used to generate cDNA libraries with M-MLV reverse transcriptase (ThermoFisher). The absolute levels of target genes were determined using the QX200™ Droplet Digital PCR system (Bio-Rad, Hercules, CA, USA). The PCR mix was prepared with 2 × QX200™ EvaGreen ddPCR Supermix. Primers used for ddPCR are listed in Supplementary Table S1. The ddPCR mix (20 µL) along with 70 µL of QX200™ Droplet Generation oil was loaded into the DG8 cartridge for droplet generation. 40 µL droplets were generated using the QX200™ Droplet Generator. Droplets were transferred to a 96-well plate, and the plate was heat-sealed and placed in C-1000 thermal cycler (Bio-Rad) for amplifying target sequences. After the PCR reaction, the 96-well plate was loaded into QX200™Droplet Reader and analyzed by Quantasoft software v1.4.0.

### 2.8. Kyoto Encyclopedia of Genes and Genomes (KEGG) and Gene Ontology (GO) Analysis

KEGG and GO analysis was performed using the web tool EnrichR (https://maayanlab.cloud/Enrichr/(accessed on 22 March 2022) [9] with KEGG 2021 human database and GO molecular function 2021 database.

### 2.9. The Cancer Genome Atlas Program (TCGA) and GTEx Data Analysis

Differential expression between normal and tumour tissues was analyzed by the web tool GEPIA2 (http://gepia2.cancer-pku.cn/#index (accessed on 12 January 2022). Pan-cancer survival analysis was performed by using the Kaplan–Meier Plotter (https://kmplot.com/analysis/index.php?p=service (accessed on 20 February 2022). All possible cut-off values between lower and higher quartiles were computed, and the best performing threshold was used as a cut-off. Patients were split by this cut-off value and follow-up threshold was 60 months. *p* value and hazard ratios were automatically calculated.

### 2.10. Enzyme-Linked Immunosorbent Assay (ELISA)

Nunc-Immuno™ MicroWell™ 96 well solid plates (Sigma, Cat# M9410-1CS) were coated with 100 µL per well of 2 µg/mL human LCN2 capture antibody (R&D systems, Cat# DY1757) in PBS and incubated at 4 °C overnight. The plates were washed three times with 300 µL washing buffer (0.05% Tween-20 in PBS, pH 7.2) per well. After each wash, the plates were tapped on a paper towel to ensure no liquid remained in the wells, followed by blocking with 300 µL blocking buffer (2% BSA in PBST, pH 7.2) per well for 1 h at RT. After washing the plates three times with 300 µL washing buffer per well, 100 µL of standards or samples (diluted in 1% BSA in PBST) were added to the wells and plates were incubated for 2 h at RT. The plates were washed 3 times with 300 µL wash buffer per well, after which 100 µL of 25 ng/mL human LCN2 detection antibody (R&D systems, Cat# DY1757) was added to each well and incubated for 2 h at RT. After washing 3 times, 100 µL streptavidin-HRP B (R&D, Cat# DY998) was added to each well and incubated for 20 min at RT, with foil covering for protection from light. The plates were washed 3 times with 300 µL washing buffer and then 100 µL of 1 mg/mL TMB (Sigma, Cat# T5525) solution. Then, 1 mg TMB in 1 mL DMSO was added to each well and incubated for 20 min at RT with foil covering. The reaction was stopped by adding 50 µL stop solution (2N H2SO4) to each well. The plates were read at wavelengths 450 nm and 540 nm.

### 2.11. Quantitative Mass Spectrometry Analysis of PC3 and PC3* Proteomes

Proteins from cells lysates of each clone of PC3 (parental, clone 1, clone 4 and clone 5) and PC3* (clone 2 and clone 3) were quantitated using a BCA kit (Thermo-Fisher Scientific) and solubilized (1% [*w/v*] sodium deoxycholate, 100 mM Tris pH 8.0) in preparation for mass spectrometry analysis. Lysates were denatured and alkylated through the addition of 5 mM Tris (2-carboxyethy) phosphine (TCEP), 20 mM chloroacetamide and incubation and vortexing for 5 min at 99 °C. Samples were digested with trypsin at 37 °C overnight. Extraction with ethyl acetate/2% formic acid (FA) was performed to remove detergent and the aqueous phase concentrated by vacuum centrifugation. Peptides were reconstituted in 0.5% FA and loaded into pre-equilibrated small cation exchange stage-tips (Empore Cation Exchange-SR Supelco Analytical). Peptides were eluted using 20% acetonitrile (ACN), 0.5% FA.

Desalted peptides were separated on a Thermo Ultimate 3000 RSLCnano UHPLC system and analyzed on aThermo Q-Exactive HF Orbitrap mass-spectrometer (Thermo-Fisher Scientific, Waltham, MA, USA). Peptides (1 µg) were loaded onto an Acclaim PepMap 100 C18 75 um × 20 mm trap column(Thermo-Fisher Scientific, Waltham, MA, USA) and washed at 6 µL/min for 6 min (Buffer C: 0.05% (*v/v*) trifluoracetic acid, 2% (*v/v*) acetonitrile) before switching the pre-column in line with the analytical column held at 55 °C, an Acclaim PepMap C18, 2 µm particle size, 75 μm ID × 75 cm length, (Thermo-Fisher Scientific, Waltham, MA, USA). The separation of peptides was performed at 250 nL/min using a linear acetonitrile gradient of buffer A (0.1% (*v/v*) formic acid, 2% (*v/v*) acetonitrile) and buffer B (0.1% (*v/v*) formic acid, 80% (*v/v*) acetonitrile), starting at 12% buffer B to 33% over 125 min, then rising to 45% B over 15 min followed by 99% B in 15 min. The column was then cleaned for 3 min at 99% B flowed by a 10 min short equilibration step completed at 1% B. Blanks with a 30 min re-equilibration were run between sample injections.

MS data was collected on an Orbitrap mass analyzer using a 180 min Data Dependent Acquisition (DDA) method. Dynamic exclusion parameters were set as follows: exclude isotope on, exclude after *n* = 1 times, duration 30 s, and used the peptide monoisotopic peak determination mode. Lock mass was set using m/z 445.12003 of polysiloxane for internal mass calibration. MS1 scans were at 60,000 resolution, m/z 350–1500, AGC target 3 × 10^6^, max injection time of 30 ms, the isolation window of the quadrupole for the precursor was 1.4 m/z. The top twelve ions were fragmented per cycle, accepted charges were 2–7. MS2 scans were at 30,000 resolution, AGC target 1 × 10^5^, injection time max 45 ms.

Raw files were processed using MaxQuant and searched against the human UniProt database (Annotation CGA_000001405.27). Principle component analysis was performed in R and figure created in Adobe Illustrator.

### 2.12. Clinical Samples

Serum samples of patients with breast, colorectal, pancreatic cancers or no cancer were obtained from the Victorian Cancer Biobank (VCB) with Human Ethics approval (HEC 18025). Serum samples were also obtained from patients with known advanced or metastatic cancer, stratified as having cachexia or not, were obtained as part of a prospective clinical trial (NCT04127981). The diagnostic criteria for cachexia are unintentional weight loss more than 5% over the previous 6 months, or more than 2% in individuals with a decreased body-mass index of <20 kg/m^2^, or skeletal muscle wasting (sarcopenia). Sarcopenia was defined as SMI < 7.26 kg/m^2^ in males and <5.5 kg/m^2^ in females, according to the European consensus on definition and diagnosis.

### 2.13. Data Presentation and Statical Analysis

Graphs were constructed with GraphPad Prism. One-way ANOVA was performed to calculate significance between three or more groups. For body weight and tumour growth curve analysis, two-way ANOVA with repeated measures was used to analyze the significance. Significant differences (*p* < 0.05) in body mass of PC3 vs. PC3* + PBS and PC3* + PBS vs. PC3* + 002, and tumour of PC3* + PBS and PC3* + 002 were observed after day 29. Only *p* values at the end point were presented in the figures. Venn diagrams were plotted with web tool http://www.bioinformatics.com.cn/srplot (accessed on 18 March 2022). Heatmaps were graphed with ggplot with hierarchical clustering and Pearson correlation.

## 3. Results

### 3.1. PC3* Preclinical Cancer Cachexia Model

To identify tumour-derived biomarker candidates, we developed a PC3* preclinical cancer cachexia model. PC3* is a subclone cell line selected from the human prostate cancer cell line PC3. Short tandem repeat (STR) profiling confirmed that the PC3* cell line is derived from the human prostate carcinoma cell line PC3. However, mass spectrometry analysis and clustering revealed distinct differences in protein expressions between parental PC3 and PC3* cells (Figure S1A). Diverse clustering was observed among different clones derived from the parental PC3 cells, whilst clones from PC3* cell line clustered closely, suggesting that the parental PC3 cell lines may include several subclones with different expression profiles. Fn14 was expressed by both cell lines (Figure S1B,C) and no significant difference was observed on growth of PC3 and PC3* cells based on a cell proliferation assay (Figure S1D).

**Figure 1:**
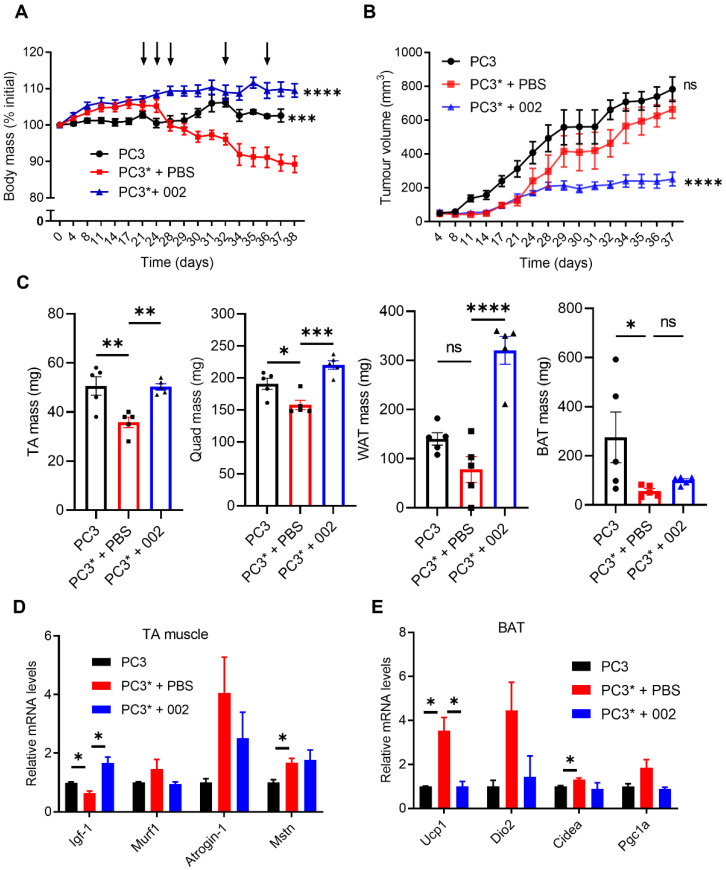
Characterization of the PC3* cancer cachexia model. (**A**) Standardized group body mass curve in PC3/PC3* models over the entire experimental period (38 days after tumour injection). Antibody treatment (↓; 10 mg/kg). *** *p* < 0.001, **** *p* < 0.0001 versus PC3* + PBS group. Data was analyzed by two-way ANOVA with repeated measures. (**B**) Tumour volume curves. Data are means ± SEM (*n* = 5 for each group). **** *p* < 0.0001 versus PC3* + PBS group. Data was analyzed by two-way ANOVA with repeated measures. (**C**) Weight of TA (left), WAT (middle), and BAT (right) from mice in the PC3* cancer cachexia model. Data are means ± SEM (*n* = 5 for each group). * *p* < 0.05; ** *p* < 0.01; *** *p* < 0.001; ns represents no significance. (**D**) RNA expression of genes related to muscle wasting in TA muscles. (**E**) RNA expression of genes related to wasting of adipose tissue in BAT. Data are means ± SEM (*n* = 3 for each group). * *p* < 0.05. Data was analyzed by one-way ANOVA.

NSG mice subcutaneously inoculated with PC3 cells did not present significant weigh loss within 40 days after inoculation (Figure 1A). In contrast, the PC3* cell line caused aggressive and rapid weight loss in the same timeframe (Figure 1A). NSG mice bearing PC3* tumors started losing weight from day 25 after inoculation of tumour cells and reached the endpoint before day 40 (Figure 1A), due to exacerbated weight loss and deteriorated body condition. Mice bearing PC3 tumors were not cachectic at the endpoint of this study (Figure 1A and Figure S2A) and served as a non-cachectic control for the PC3* mice. Tumour sizes were similar for PC3 and PC3* tumors (Figure 1B and Figure S2B).

In a previous study, we found that an anti-Fn14 antagonistic monoclonal antibody (002 mAb) could prevent cancer cachexia in several preclinical models [7]. To test the anti-cachexia effect of 002, mice bearing PC3* tumors were treated with 002 mAb from day 21. Compared with a PBS control, 002 mAb significantly maintained body weight of mice bearing PC3* tumors (Figure 1A and Figure S2A). Mice bearing PC3* tumors had significant loss of muscle and adipose tissues and 002 mAb treatment prevented these losses (Figure 1C). Muscle fibers were significantly smaller in PC3* cachectic mice, and treatment with 002 mAb prevented muscle fiber shrinkage caused by the PC3* tumors (Figure S2D–F). Muscle RING-finger protein-1 (MuRF1) and muscle specific F-box protein (Atrogin-1) are two ubiquitin ligases that have been shown to be upregulated in cachectic muscles from some cancer cachexia murine models [10,11]. Nevertheless, a recent study has shown that expression of MuRF1 and Atrogin-1 was not altered in muscle from cachectic patients [12]. Consistent with these findings, no significant change on expression of MuRF1 and Atrogin-1 was observed in tibialis anterior (TA) muscle in the PC3* model (Figure 1D). Interestingly, IGF-1, a factor promoting muscle protein synthesis, was downregulated in muscle tissues from mice bearing PC3* tumors and upregulated in mice treated with 002 mAb (Figure 1D). This finding suggests that reduced protein synthesis in muscle cells could contribute to the wasting symptoms in cancer cachexia. Muscle myostatin (MSTN) expression is upregulated in some preclinical cancer cachexia models, which could contribute to increased muscle protein breakdown [13,14]. Expression of myostatin was significantly higher in TA muscle from mice bearing PC3* cachectic tumors (Figure 1D). However, cachectic mice treated with 002 mAb had similar levels of myostatin to PBS treated cachectic mice, suggesting that myostatin signaling may not play a key role in muscle wasting of PC3* model. To investigate the wasting of adipose tissues in PC3* mice, expression of genes involved in thermogenesis and lipolysis were measured in both white adipose tissue (WAT) and brown adipose tissue (BAT). Increased expression of Uncoupling protein 1 (UCP1) in BAT was observed in cachectic mice, which was downregulated in mice treated with 002 mAb (Figure 1E). Although 002 mAb treatment reduced the expression of UCP1 and Iodothyronine Deiodinase 2 (DIO2), expression of the two genes in WAT was not higher in cachectic mice than non-cachectic mice (Figure S2C). Compared with the PC3* cachectic group, treatment with 002 mAb significantly increased the mass of WAT. The reduced expression of browning and thermogenesis proteins UCP1 and DIO2 may contribute to increased WAT mass in 002 mAb treated mice. Cell death-inducing DNA fragmentation factor-alpha-like effector A (CIDEA) and Peroxisome proliferator-activated receptor gamma coactivator-1-α (PGC-1α) have been shown to be involved in regulating wasting and browning of adipose tissues in cancer cachexia [15,16]. Increased CIDEA expression was observed in BAT from PC3* model, but no significant change was observed with 002 mAb treatment (Figure 1E). There was also no obvious difference on PGC-1α levels across all groups (Figure 1E).

Treatment with 002 mAb significantly reduced PC3* tumour sizes in vivo (Figure 1B and Figure S2B). The in vitro cell proliferation study found that 002 mAb inhibited tumor growth after treatment of 96 h (Figure S1D), suggesting a direct tumour inhibiting effect of 002 mAb. The anti-cancer effect of monoclonal antibodies targeting Fn14 has been reported in some studies [17,18], whilst our previous study suggests that the anti-cachexia effect of 002 mAb was independent of its anti-cancer effect [7]. The observed anti-tumour effect of 002 mAb in the PC3* model, could contribute to its anti-cachexia effect. The involvement of a PC3 non-cachectic control, however, ensures that we were not identifying anti-cancer biomarkers, as PC3 and PC3* tumors have similar sizes. Overall, we established a novel preclinical cancer cachexia model that could be utilized to screen biomarkers for cancer cachexia.

### 3.2. Screening of Cachexia Related DEGs Based on RNA-Seq

**Figure 2:**
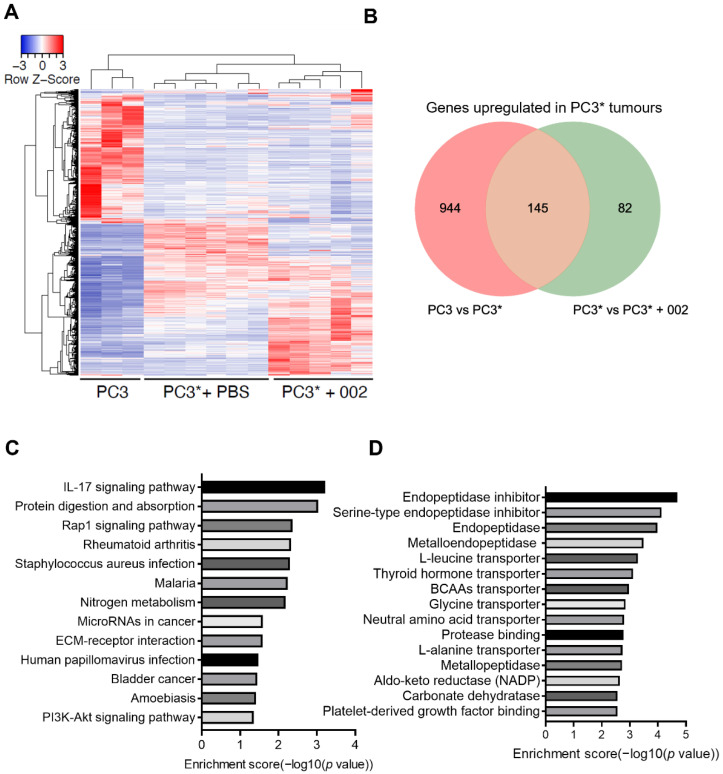
Screening DEGs upregulated in cancer cachexia. (**A**) Heatmap of RNA-sequencing results for PC3 (*n* = 3), PC3* + PBS (*n* = 6), and PC3* + 002 (*n* = 5) groups. (**B**) Venn diagram of DEGs of PC3 vs. PC3* and PC3* vs. PC3* + 002. (**C**) KEGG pathway analysis of tumour DEGs upregulated in cancer cachexia and downregulated by 002 mAb. (**D**) Gene Ontology Molecular Function analysis of tumour DEGs upregulated in cancer cachexia and downregulated by 002 mAb.

Tumors were collected from three groups of mice: PC3 (non-cachectic), PC3* + PBS (cachectic), and PC3* + 002 mAb (non-cachectic). Subsequently, total RNA was extracted and sequenced by using Illumina NextSeq 500 platform. More than 20 million reads for each sample were obtained after RNA-Seq. A total of 14,943 genes were detected after excluding genes with less than 1 Reads Per Kilobase of transcript per Million mapped reads (RPKM). Multidimensional scaling (MDS) analysis confirmed near identical clustering for individual mice within groups, suggesting the model is highly reproducible and each group is distinct from one another (Figure S3A). Fold change ≥ 2 and *p* value ≤ 0.05 was considered as the threshold of DEGs. More than 1800 DEGs were observed between PC3 and PC3* group, and 688 DEGs were identified between PC3* and the PC3* treated with the 002 antibody (Figure 2A and Figure S3B). The main intention of this study was to identify biomarkers that are positively correlated with cancer cachexia. Therefore, only DEGs that were upregulated in PC3* + PBS tumors were selected. To exclude the possibility that the biomarkers were related to tumour growth, both PC3 and PC3* + 002 tumors were included for comparison. A total of 145 DEGs were left and identified as biomarker candidates for further study (Figure 2B). These DEGs were all upregulated in cachectic tumors, and responsive to 002 mAb treatment.

To reveal a potential mechanism of how Fn14 induced, and 002 mAb prevented cancer cachexia, enrichment analysis was performed to search DEG related pathways. The DEGs were enriched in several signaling pathways (*p* < 0.05), including IL-17, Rap1, PI3K-AKT signaling pathways (Figure 2C). Although TWEAK, the only known ligand of Fn14, was shown not to be required for the production of cachexia in preclinical models [7], it has previously been shown to induce the production of IL-17 and that the blockade of Fn14, by adding Fn14-Fc to cell culture medium, could effectively inhibit the production of IL-17 [19]. Overexpression of IL-17 has been shown to induce cachexia in mice through Toll-like receptor 4 signaling in muscle tissues [20]. Rap1 signaling is involved in tumour cell migration, invasion and metastasis [21]. However, a correlation between the Fn14 and Rap1 signaling pathway has not been reported. TWEAK-Fn14 has also been shown to activate the PI3K-AKT pathway, although the detailed mechanism is not known [22]. It was expected that the antagonistic anti-Fn14 antibody could downregulate this pathway. However, whether these tumoral pathways are related to the production of cachexia-inducing factors in cancer cachexia is still unknown.

Gene Ontology (GO) analysis of molecular function revealed that many DEGs were related to the regulation of endopeptidase activity and amino acid transmembrane transporter activity (Figure 2D). This supports data that endopeptidases such as asparagine endopeptidase and metallo-endopeptidases are associated with tumour invasion and metastasis [23,24,25], although these studies did not extend to the role of endopeptidases in cachexia. The SLC36A1, SLC7A5, SLC7A8 genes, which are related to amino acid transmembrane transporter activity, could play a role in tumour uptake of amino acids produced by host protein degradation during cancer cachexia and contribute to the growth of tumors.

### 3.3. Secretomic and Transcriptomic Analysis of Cachexia Related DEGs

**Figure 3:**
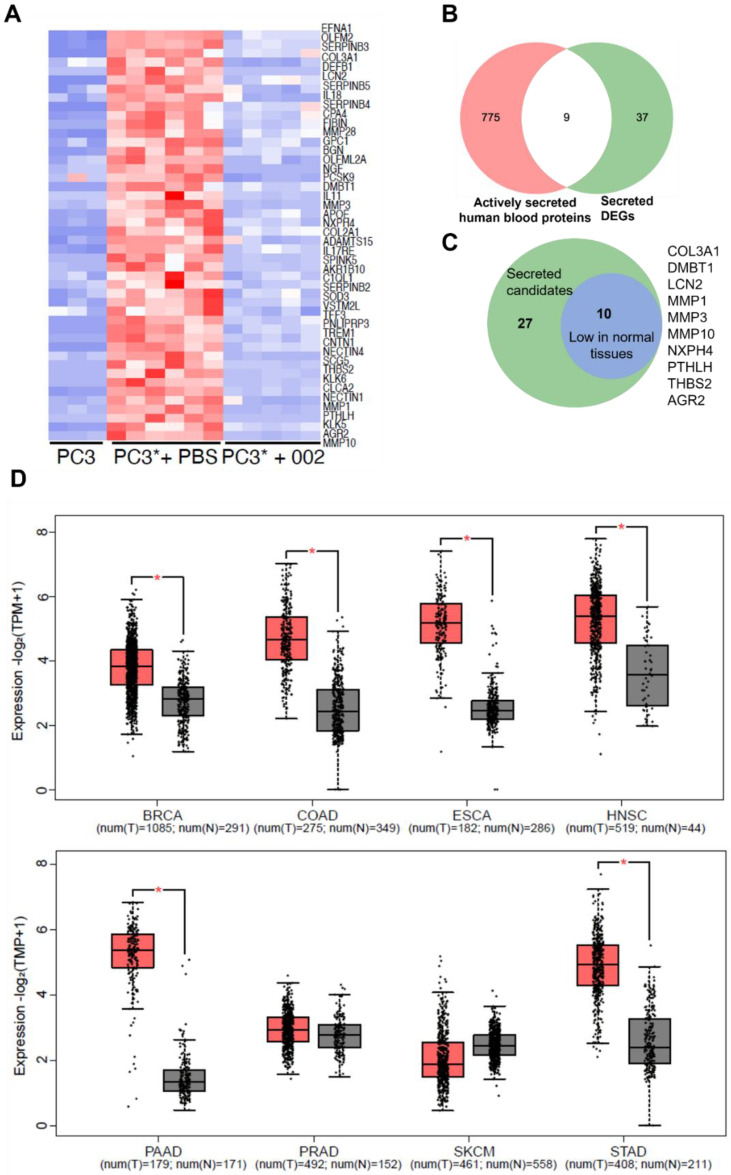
Secretomic and transcriptomic analysis of cachexia related DEGs using online databases. (**A**) Heatmap of DEG candidates predicted to be secreted. (**B**) Venn diagram of secreted DEG candidates and proteins actively secreted to blood in normal states. (**C**) Venn diagram of secreted DEGs and candidates which have low expression in normal tissues. (**D**) Transcriptomic analysis of combined expression of 10 biomarker candidates in 8 cancers compared with normal tissue gene expressions with TCGA and GETx databases. Red box and gray box indicate tumour and normal tissues, respectively. * *p* > 0.05. Numbers of patients in each group are noted in each graph. BRCA, breast invasive carcinoma; COAD, colon adenocarcinoma; ESCA, colon adenocarcinoma; HNSC, head and neck squamous cell carcinoma; PAAD, pancreatic adenocarcinoma; PRAD, prostate adenocarcinoma; SKCM, skin cutaneous melanoma; STAD, stomach adenocarcinoma.

Although tumour biopsies have been widely applied in clinical diagnosis, measurement of circulating biomarkers would be preferred. Compared with tumour biopsy, blood samples are more accessible and less invasive. Additionally, in the situation of monitoring a treatment, the taking of tumour biopsies are generally not an option, as the tumour may have metastasized, or it is impractical to constantly obtain tumour samples during treatment. To identify secreted proteins that could act as circulating biomarkers, we performed in silico secretomic analysis on all selected DEGs. By selecting for proteins with signal peptides, and excluding proteins with transmembrane helices, 46 candidates were predicted to be secreted (Figure 3A). Transcriptional levels of some candidates in tumors from the PC3* model was analyzed to confirm the accuracy of the sequencing results (Figure S4). RNA levels measured by ddPCR aligned with the expression shown by RNA-seq, supporting the accuracy and reliability of the RNA-seq data.

However, the fact that these proteins are predicted to be secreted is not enough to justify their use as putative cancer cachexia biomarkers, as the above analysis does not consider that they may be highly expressed and secreted by normal tissues. Circulating levels of such biomarkers could be largely derived from host tissues, but whether these biomarkers are upregulated in cachectic host tissues is unknown. Using the Human Protein Atlas, some candidates were predicted as proteins actively secreted to human blood in the normal state [26], including SCG5, IL11, IL18, PCSK9, TFF3, EFNA1, TREM1, APOE, SOD3 (Figure 3B). Ideal tumoral biomarkers for cancer cachexia would be expected to be highly expressed in tumors which cause cachexia, but low in normal tissues. To examine expression of these candidates, we mined RNA-seq and microarray data from The Cancer Genome Atlas (TCGA) and the Genotype-Tissue Expression (GTEx) databases, by using Gene Expression profile Interactive Analysis (GEPIA) web tool [27]. Given the fact that cachexia is presented in many cancers, genes not differentially expressed between cancers and most normal tissues, may not be ideal biomarkers, and thus were removed (Figure S5). Eventually, 10 candidates were chosen as the most promising biomarkers (Figure 3C): COL3A1, DMBT1, LCN2, MMP1, MMP3, MMP10, NXPH4, PTHLH, THBS2, AGR2. The combined expression signature of these biomarkers and expression of individual biomarkers in most cancer tissues is higher than normal tissues (Figure 3D and Figure S6).

### 3.4. The Tumour Expression Profile of Potential Biomarkers in Different Cancers Is Correlated with Risk of Cachexia and Cancer Outcomes

**Figure 4:**
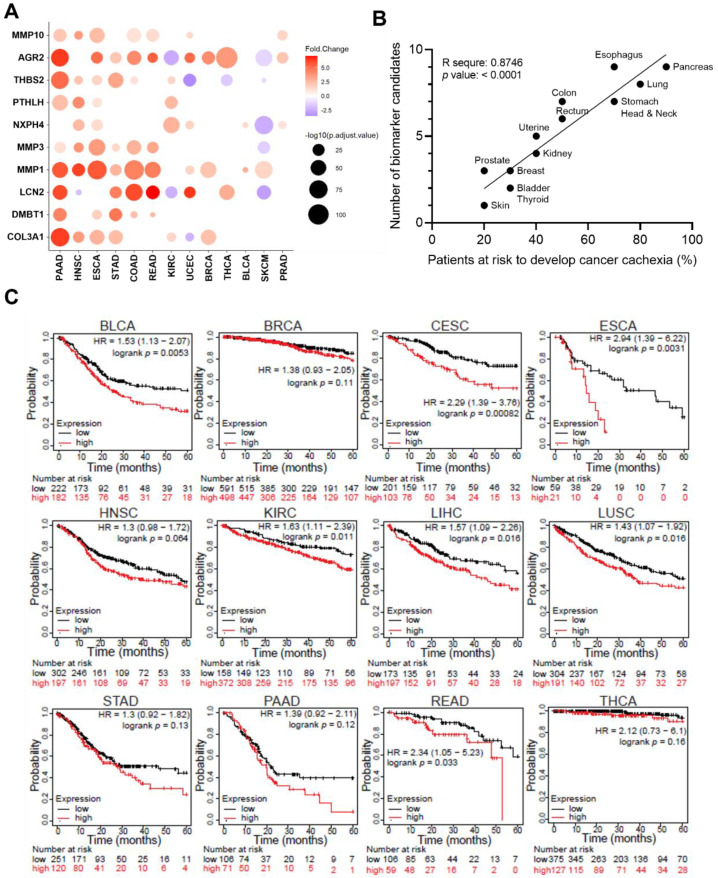
Expression of biomarker candidates in different cancers and survival analysis. (**A**) Schematic representation of expression patterns of all 10 biomarker candidates in different cancers of TCGA. Differential expression levels were calculated using web tool GEPIA2 by comparing tumour and normal tissues from TCGA and GETx databases. Upregulated and downregulated genes with absolute values of fold change > 2.0 and q value < 0.01 (ANOVA) are shown in red and blue, respectively. The size of the circles represents -log10(adjust *p* value). (**B**) Linear regression analysis of the number of upregulated biomarker candidates and risk to develop cachexia in different cancer types. Pearson’s correlations coefficient (r) with corresponding *p* values are calculated by GraphPad Prism. (**C**) Kaplan–Meier Plotter of survival for 10 biomarker expression signatures in different cancers. Patient numbers, HR value, and *p* values are noted in the graphs. BLCA, bladder urothelial carcinoma; BRCA, breast invasive carcinoma; CESC, Cervical squamous cell carcinoma and endocervical adenocarcinoma; COAD, colon adenocarcinoma; ESCA, Esophageal carcinoma; KIRC, kidney renal clear cell carcinoma; HNSC, head and neck squamous cell carcinoma; LIHC, liver hepatocellular carcinoma; LUSC, lung squamous cell carcinoma; PAAD, pancreatic adenocarcinoma; PRAD, prostate adenocarcinoma; READ, rectum adenocarcinoma; SKCM, skin cutaneous melanoma; STAD, stomach adenocarcinoma; THCA, thyroid carcinoma; UCEC, Uterine Corpus Endometrial Carcinoma.

Although biomarker candidates were highly expressed in many individual cancer types, it is important for them to correlate collectively to predict cancer cachexia. Cancer cachexia is widely present in different cancers, with a high prevalence in cancers such as pancreatic, gastro-esophageal, head and neck cancers [28,29]. Interestingly, we found that expression profiles of potential biomarkers were distinct in different tumour types (Figure 4A). Pancreatic adenocarcinoma (PAAD) showed the highest number (9 out of 10) of upregulated biomarkers, while skin cutaneous melanoma (SKCM) had only one upregulated biomarker—MMP1. In fact, MMP1 is upregulated in most cancer tissues, regardless of the prevalence of cachexia in the specific tumour types. AGR2 was the other biomarker that appeared upregulated in most cancers. However, distribution of other biomarkers in all cancer types were not identical. Overall, most biomarkers were upregulated in cancers such as PAAD, stomach adenocarcinoma (STAD), Esophageal carcinoma, (ESCA), head and neck squamous cell carcinoma (HNSC), whereas only a few biomarkers were upregulated in prostate adenocarcinoma (PRAD; NXPH4, AGR2, MMP10), breast invasive carcinoma (BRCA; COL3A1, MMP1, AGR2), bladder urothelial carcinoma (BLCA; MMP1, NXPH4), thyroid carcinoma (THCA; LCN2, AGR2) (Figure 4A).

Since the risk of developing cancer cachexia has been well defined [30], we then explored the total number of upregulated biomarkers in each tumour type and their correlation with the risk of developing cancer cachexia. Although the datasets from TCGA may not fully represent the differences in severity of cachexia in these cancer types, they have the potential to provide relevant frequency data. The total number of upregulated biomarkers is strongly aligned with the risk of cachexia in each cancer types with R square value of 0.8746 (*p* < 0.0001) (Figure 4B). Together, these findings suggested that the expression profiles of these biomarker candidates could potentially predict the incidence of developing cancer cachexia.

Cancer patients with cachexia generally have worse outcomes and survivals than patients without cachexia, in part due to the intolerance of anti-cancer treatments such as chemotherapy and radiotherapy. We therefore determined if expression of these biomarker candidates is related to the survival of cancer patients. In the overall cancer patient populations, patients with lower levels of biomarker candidates have significantly longer survival (Figure S7A), with a hazard ratio (HR) of 2.3. Using the Kaplan–Meier Plotter for pan-cancer, correlation between survival and expression signature of the 10 biomarker candidates was analyzed. High expression of biomarkers is correlated with decreased overall survival in most cancer types listed in the database (Figure 4C). Using the 10 biomarkers, we also identified biomarkers that have higher relevance in predicting decreased survival of individual cancer types (Figure S7C). Prognosis of each cancer type was correlated with different biomarker candidates. In PAAD, high expression of PTHLH, MMP3, MMP1, MMP10 and THBS2 was significantly correlated with poor survival, whereas only MMP1 and NXPH4 are associated with low survival in BRCA patients (Figure S7C). Overall, these findings suggest that the biomarker candidates could be prognostic markers to predict survival outcomes.

### 3.5. Validation of Circulating LCN2 as a Biomarker for Cancer Cachexia

**Figure 5:**
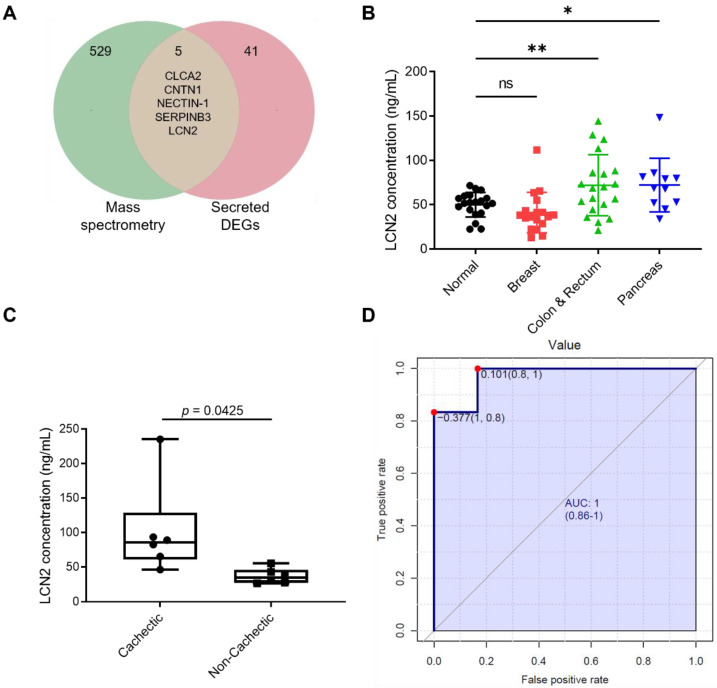
Validation LCN2 as a biomarker for cancer cachexia. (**A**) Venn diagram of human proteins detected in mouse plasma from the PC3* model by mass spectrometry and secreted DEG candidates. (**B**) ELISA results of LCN2 levels in serum samples from normal patients and patients with breast, colorectal, or pancreatic cancer. Data are mean ± SEM (*n* = 20 for normal; *n* = 18 for breast; *n* = 20 for colon & rectum; *n* = 11 for pancreas). Data was analyzed by one-way ANOVA with Fisher’s LSD using a false discovery rate of 0.05. * *p* < 0.05; ** *p* < 0.01; ns means no significance. (**C**) ELISA results of circulating LCN2 levels from cancer patients with and without cachexia (*n* = 6 for each group). (**D**) ROC curve analysis of LCN2 level in cancer patients with and without cancer cachexia.

More than 90% of proteins in serum/plasma are albumins and globulins, while less than 1% are low-abundant proteins [31]. Identification of low-abundant proteins in serum/plasma samples generally requires depletion of abundant proteins at the beginning of the pipeline [32,33]. To evaluate if the biomarker candidates were presented in plasma, mass spectrometry was employed to analyze plasma proteins in the PC3* preclinical model after removal of abundant proteins. Although more than 500 human proteins were detected, only 11 were also detected using RNA-Seq, conforming to the criterion that they were highly expressed in cachectic tumors and downregulated by 002 mAb. Interestingly, six of these are not secreted proteins, including CASP14, KRT14, KRT16, KRT5, KRT6A and TNS4. Many of these may be released to plasma via extracellular vesicles (EVs), but keratins are more likely to be common contaminants of mass spectrometry [34]. High expression of TNS4 in tumors was shown to associate with poor survival in different cancers [35,36]. Nevertheless, to our knowledge, correlation of circulating TNS4 with cancer cachexia has not previously been reported. The five others, including CLCA2, CNTN1, NECTIN-1, SERPINB3 and LCN2, were deemed putative secreted biomarker candidates (Figure 5A). In another mass spectrometry analysis of plasma samples from the previously published mouse MEF v12 Hras Fn14 model, LCN2 was exclusively detected in plasma samples from cachectic mice (Supplementary Data). Western blotting of serum samples from PC3* model showed high level of LCN2 in samples from mice bearing the PC3* tumors, compared with samples from the PC3 group (Figure S8). Moreover, 002 mAb treatment reduced serum LCN2 level in mice bearing PC3* tumors. Other biomarker candidates were not identified by mass spectrometry; however, this does not discount that these proteins are potential biomarkers for cancer cachexia.

High level of LCN2 was correlated with low survival in all cancer patients (Figure S7B). To confirm if LCN2 can be used as a potential biomarker for diagnosing human cancer cachexia, we examined LCN2 levels in serum samples from patients with different cancers. Patient serum samples were obtained from the public Victorian Cancer Biobank and we chose samples from patients with breast, colorectal, pancreatic cancer, and control patients without cancer. Details of weight loss or body composition were not available for these patients. However, the choice of these groups was based on the overall prevalence of cachexia in different cancers, which has been previously reported [29,37], with high rates in pancreatic cancer, intermediate levels in colorectal cancers, and low incidence in breast cancer. The circulating LCN2 levels in these cancer patients were determined using a capture/reporter ELISA. Compared with normal non-cancer and breast cancer patients, patients with colorectal or pancreatic cancer had significantly higher levels of circulating LCN2 (Figure 5B). No significant difference in LCN2 levels was observed between normal non-cancer patients and patients with breast cancer (Figure 5B).

To further assess the feasibility of LCN2 as a biomarker for diagnosing cancer cachexia, serum samples were obtained from a cancer cachexia clinical trial (NCT04127981). This ongoing trial includes patients with pancreatic and colorectal cancers. All patients had advanced disease and received chemotherapy with or without targeted therapy. The cachectic state of each patient was recorded, including body composition (DEXA) (Tables S2 and S3), and a blind measurement of circulating serum LCN2 was performed. Compared with cancer patients with no cachexia, cachectic cancer patients had significantly higher level of LCN2 (Figure 5C). To further determine whether changes in circulating LCN2 levels could serve as a biomarker of cancer cachexia, Receiver Operating Characteristic (ROC) analysis was performed by using the Biomarker Analysis feature of MetaboAnalyst [38]. The analysis examined the ability of a given target to distinguish two groups of patients, in this case, cachectic and non-cachectic patients. LCN2 was able to significantly separate cachectic patients, with an area under the ROC curve (AUC) of 0.972 (95% CI 0.833–1, *p* = 0.004646 after data log transformation and scaling) (Figure 5D). Taken together, these findings suggest that LCN2 could be a valuable biomarker for cancer cachexia.

## 4. Discussion

Cancer cachexia has been an underestimated clinical problem, compromising anti-cancer treatments, significantly impacting on the quality of life, and shortening lives. Currently, many studies are solely focused on developing anti-cancer treatments, without appreciating that treating cachexia could also significantly improve the survival of cancer patients, particularly for those with late-stage cancers. Deterioration of physical fitness can result from various conditions, such as sarcopenia, anorexia, and cachexia. Although these conditions share several similar features such as muscle wasting and weakness, they should be considered as separate disorders and treated as such [39]. Anorexia and cachexia can present simultaneously in some cancer patients, and both can contribute to weight loss. Thus, treatments to increase appetite have been shown to increase body mass in some clinical studies, while failing to restore muscle function [3]. These findings imply that simply increasing food intake may not completely stop or reverse cancer cachexia, as the loss of body mass in cancer cachexia is a result of not only reduced caloric intake but also altered metabolism.

Recently, some studies have been performed to develop therapies against cancer cachexia, by targeting cachexia-inducing factors [40]. Our previous study found that tumoral Fn14 is an attractive target for treating cancer cachexia, and antagonistic anti-Fn14 monoclonal antibodies have shown a potent effect on ameliorating cancer cachexia in different preclinical models [7], which was also validated in this study. We have also developed humanized versions of the anti-Fn14 antagonistic mAb, for evaluation in the clinic. To effectively assess the outcomes of clinical studies for cancer cachexia, the use of biomarker-based assays in addition to current measurements have significant potential benefits. This would particularly be the case for slow-acting treatment, as current measurements may not be sensitive enough to detect minor improvements over a short period of time.

To identify potential biomarkers for cancer cachexia, we have established a novel human xenograft cancer cachexia model. The advantage of this model is that it includes a non-cachectic tumour control with similar genetic background to the cachectic tumour. Identification of biomarkers for cancer cachexia is difficult with the widely used C26 model, as the model does not include a non-cachectic tumour control. Additionally, a recent study has shown that the wasting mechanisms in the C26 model are distinct from human cancer cachexia, making it a potentially inappropriate human cancer cachexia model [12]. The other advantage of our PC3* model, compared with C26, is that proteins derived from the human tumour can be clearly differentiated from host murine proteins. Using RNA-seq and in silico secretomic analysis, we have identified a list of genes that were upregulated in cancer cachexia and downregulated by anti-Fn14 mAb treatment. Some candidates could be potential biomarkers for analysis in tumour biopsies and blood samples. Multiplex cytokine arrays are commonly used to screen cytokine biomarkers for different diseases [41,42]. As most biomarker candidates identified here are not cytokines, such assays are likely to be of limited use for diagnostic purposes in cancer cachexia. One possible use for biomarker analyses is to develop a customized and sensitive multiplex array that can be used to detect and measure these biomarkers simultaneously in the future.

Although high expression levels of these biomarkers in tumors were shown in datasets from TCGA and GTEx, it is unlikely that all biomarkers are upregulated in blood. Normal tissues could also actively secrete a significant number of these candidates, which may invalidate their diagnostic value for cancer cachexia. After removal of candidates that are secreted in blood in a healthy state and candidates highly expressed in normal tissues, we obtained 10 of the most promising biomarkers. The expression level of these biomarkers correlated with survival in pan-cancer analysis. One of the biomarkers—LCN2 was detected in plasma samples from cachectic mice. Subsequent analysis found that circulating LCN2 was also significantly upregulated in human cachectic patients.

The initial intention of this study was to identify both biomarkers and tumour-derived mediators that induce cancer cachexia. Although we did not examine the effect of individual biomarkers on inducing cancer cachexia, some studies have shown that several candidates in the list could promote wasting symptoms in preclinical models. PTHrP, the protein encoded by PTHLH, was shown to be secreted by tumors and trigger browning of adipose tissue in cancer cachexia [43]. Another study found that serum PTHrP levels could predict weight loss in cancer patients [44], which aligns with our findings. Tumour derived MMP1 was shown to induce ECM disruption in fat body and muscle in a drosophila cancer model, leading to muscle wasting [45], however, whether it promotes mammalian cancer cachexia is yet to be investigated. Increased circulating LCN2 levels were reported in murine pancreatic cancer cachexia models, which potentiated muscle and fat wasting in these models [46]. Further studies are required to assess whether any of the biomarkers released by the tumour are involved in inducing cancer cachexia.

## 5. Study Limitations

Although we were able to show increased serum LCN2 level in preclinical models for cancer cachexia, there are several limitations to the current study. In the control, we used PBS rather than IgG. This was based on our previous study where we showed that there were no significant differences between PBS and IgG [7] in a control group of mice with cachexia in another preclinical cachexia model. Further validation of this question will be beneficial for the PC3* cachexia model. The current study did not look at the correlation between LCN2 level and severity of cancer cachexia, a study we will explore in the future, as will the association of LCN2 levels to body weight loss, and to muscle and fat mass. The clinical validation of LCN2 as a biomarker for cancer cachexia was a preliminary study limited by the small sample size of only *n* = 6 for both cachectic and non-cachectic groups. Due to the small size of patient groups, we were not able to match the gender of the groups. All patients in the non-cachectic group were females (6 out 6), and most patients in the cachectic group were males (5 out of 6). We believe that since males have higher threshold on sarcopenia than females, explains why the SMI was not significantly different between cachectic and non-cachectic patients. All non-cachectic patients had colorectal cancer, while cachectic patients had pancreatic cancer. Although we cannot, at this stage, exclude a potential impact of cancer types on LCN2 levels, the evaluation of human cancer patients using samples from the VCB biobank samples showed no significant differences in LCN2 levels between overall colorectal and pancreatic patients (Figure 5B).

## 6. Conclusions

The results of this study suggest that several proteins could be promising biomarkers of cancer cachexia. Importantly, these biomarkers are responsive to a novel anti-cachexia therapy—anti-Fn14 mAb treatment. The signatures of the 10 most promising biomarkers are associated with survival in a pan-cancer analysis. Increased circulating levels of LCN2, one of the biomarker candidates, is associated with cachectic state in both preclinical and clinical samples. Additional studies investigating circulating levels of other biomarker candidates would be valuable to confirm their suitability for diagnosing cancer cachexia in the clinic. Overall, the findings in this study could facilitate the improved diagnosis and assessment of cancer cachexia in the future.

## 7. Patents

The authors hold a provisional patent “Methods of predicting a wasting disorder and response to treatment thereof”.

## Data Availability

Data presented in this research paper will be made available upon request to the corresponding authors.

## Supplementary Materials

The following supporting information can be downloaded at: https://www.mdpi.com/article/10.3390/cancers14225533/s1, Figure S1: Analysis of PC3 and PC3* cells; Figure S2: Body mass, tumour volume and muscle analysis of PC3* model at endpoint; Figure S3: RNA-sequencing analysis of tumors from PC3* models; Figure S4: ddPCR measurement of secreted DEGs in tumors from PC3* model; Figure S5: Transcriptomic analysis of some of the biomarker candidates highly expressed in normal tissues; Figure S6: Transcriptomic analysis of biomarker candidates highly expressed in tumor tissues compared with normal tissues; Figure S7: Correlations between levels of biomarker candidates and survival in cancer patients; Figure S8: Western blotting of LCN2 in serum samples from PC3* model; Table S1: ddPCR primer sequences; Table S2: Clinical characteristics of cohorts in clinical trial NCT04127981; Table S3: Detailed information for individual patients in clinical trial NCT04127981.
