# Supplementary material for: Identification of Potential Biomarkers for Cancer Cachexia and Anti-Fn14 Therapy
Source: Cancers (Basel). 2022 Nov 10;14(22):5533. doi: 10.3390/cancers14225533 (PMC9688504; doi:10.3390/cancers14225533)
Supplement: Supplementary file 1 [file cancers-14-05533-s001.zip › cancers-1965376-supplementary.pdf]

cancers

MDPI

Supplementary Material

Identification of Potential Biomarkers for Cancer Cachexia and Anti-Fn14 Therapy

Zhipeng Cao [1,2,†], Ingrid J. Burvenich [2,†], Kening Zhao [1,3], Clare Senko [2], Jason Glab [1,‡], Renee Fogliaro [4], Zhanqi Liu [2], Irvin Jose [1], Hamsa Puthalakath [1], Nick J. Hoogenraad [1,2], Laura D. Osellame [1,2,*] and Andrew M. Scott [2,4,5,*]

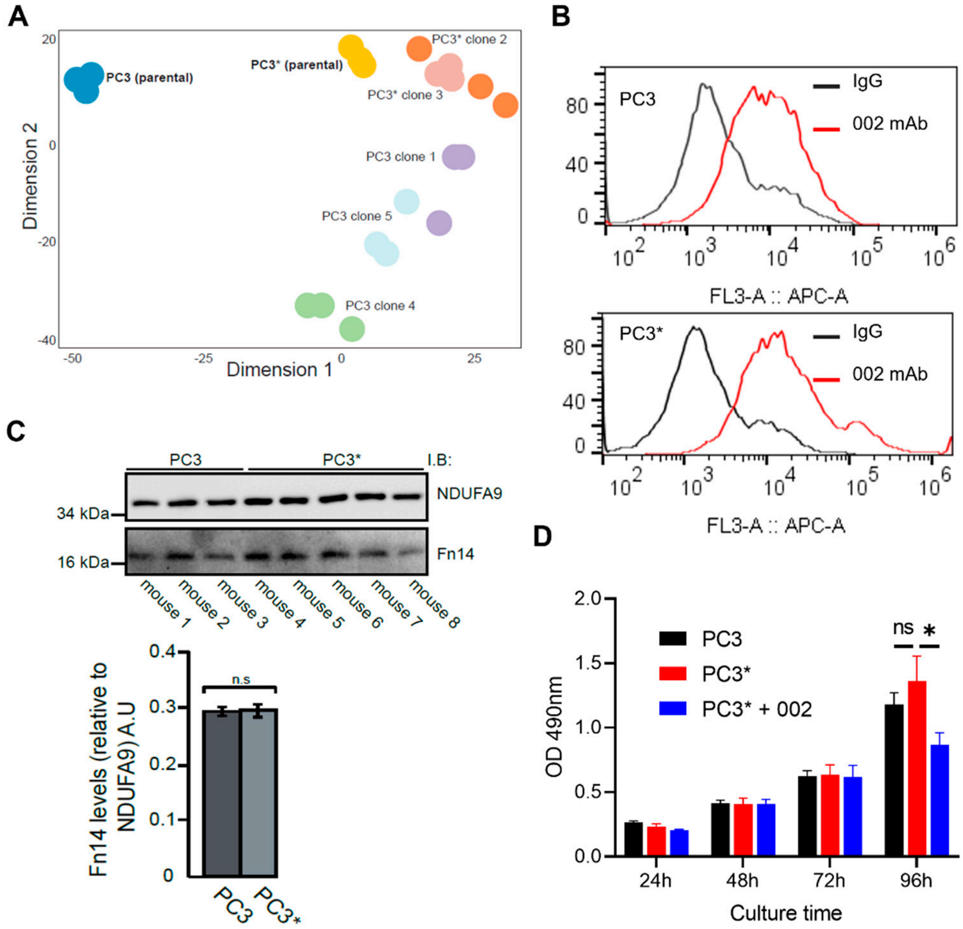

Figure S1. Analysis of PC3 and PC3* cells. **(A)**Mass spectrometry clustering (principal component analysis) of parental and subclone cell lines from PC3 and PC3* cells. **(B)** FACS analysis of Fn14 expression on PC3 and PC3* cells. **(C)** Western blotting analysis of Fn14 expression in tumour lysates of PC3 and PC3* tumors. NDUFA9 was used as a loading control. Quantification of band intensity was presented in the bar graph. Data are mean ± SEM (n = 3 for PC3 group; n = 5 for PC3* group. n.s. represents no significance calculated by Student's t-test. **(D)** Cell proliferation of PC3, PC3* and PC3* cells treated with 002 mAb. Data are mean ± SEM (n = 6 for each group). *p < 0.05 calculated by one-way ANOVA.

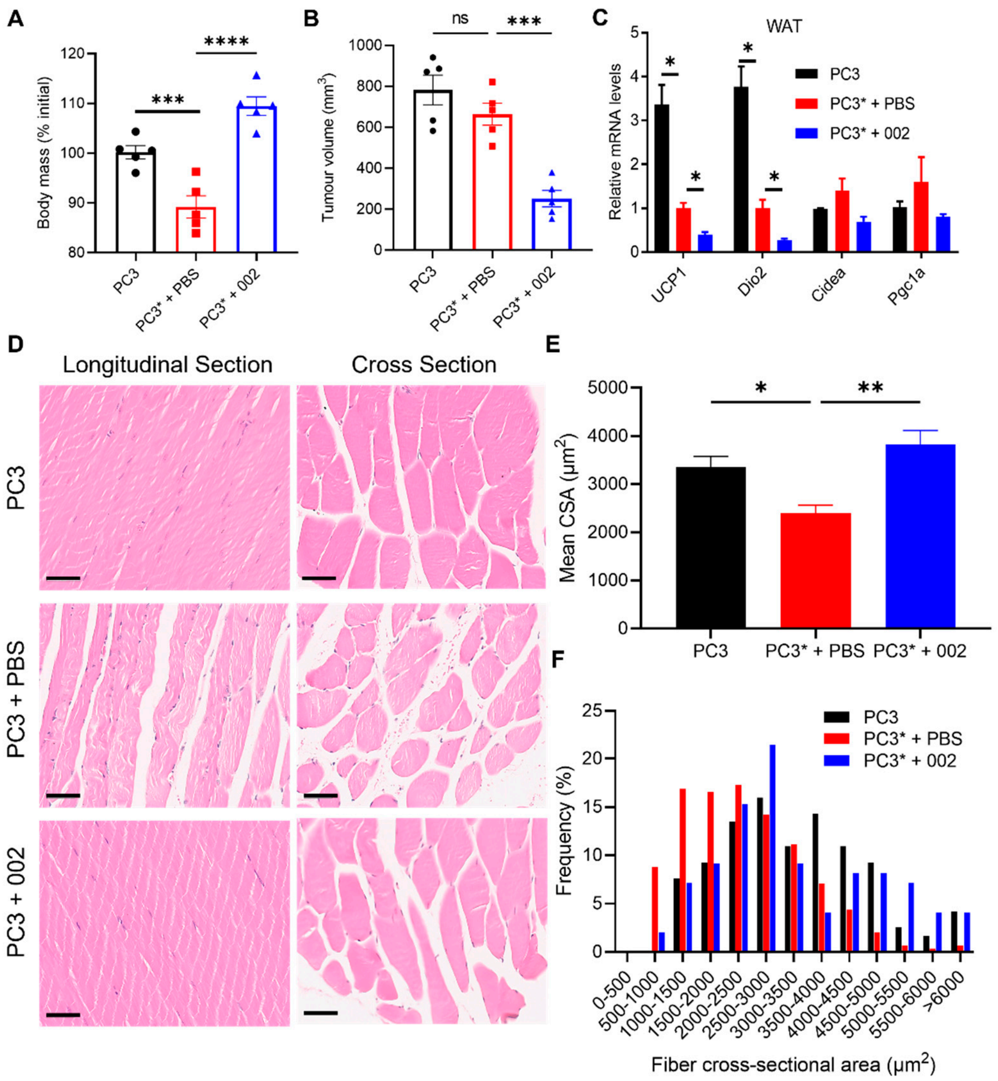

Figure S2. Body mass, tumour volume and muscle analysis of PC3* model at endpoint. **(A)** Body mass of mice bearing PC3, PC3* + PBS, and PC3* + 002 tumors. **(B)** Tumour volume of PC3, PC3* + PBS, and PC3* + 002 tumors. Data are mean ± SEM (n = 5 for each group). $^{**}p < 0.01$, $^{***}p < 0.001$, $^{****}p < 0.0001$, ns represents no significance, as calculated by one-way ANOVA. **(C)** Expression of genes related fat wasting in WAT. Data are mean ± SEM (n = 5 for each group). $^{*}p < 0.05$. **(D)** H&E staining of quadricep muscles from PC3 model. Scale bar = 50 μm. **(E)** Mean muscle fiber CSA. n = 3, $^{*}p < 0.05$, $^{**}p < 0.01$ as calculated by one-way ANOVA. **(F)** Frequency of muscle fibers at different sizes. Measurement was performed by ImageJ.

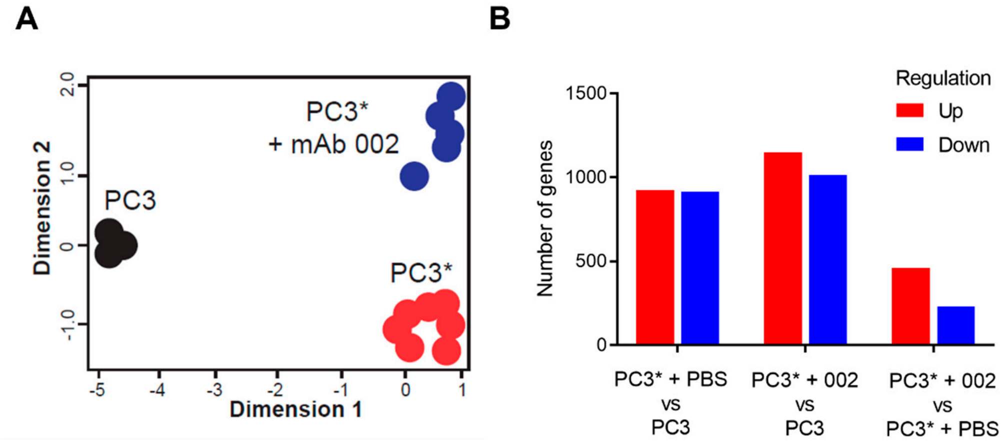

Figure S3. RNA-sequencing analysis of tumors from PC3* models. **(A)** Multidimensional scaling (MDS) analysis of sequencing data from three groups. **(B)** Upregulated and downregulated DGEs between groups.

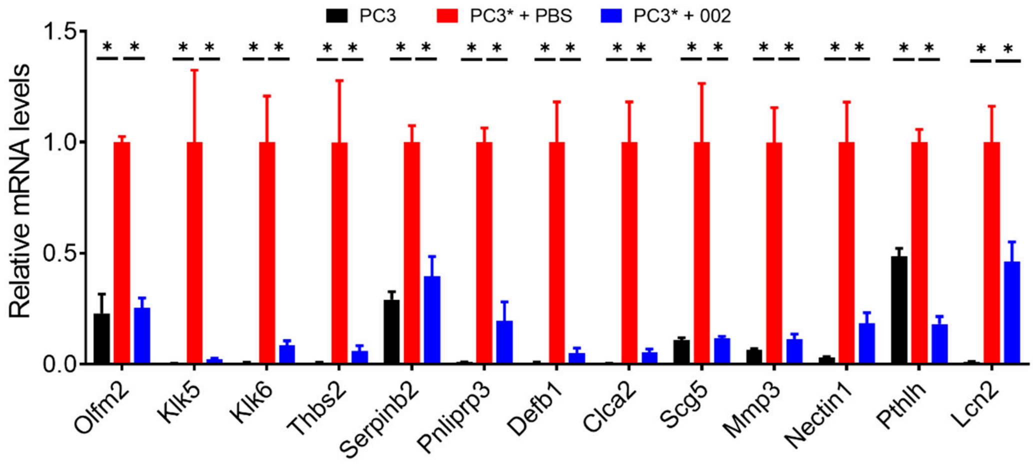

Figure S4. ddPCR measurement of secreted DEGs in tumors from PC3* model. Data are mean ± SEM (n = 3 for each group). *p < 0.05 in one-way ANOVA.

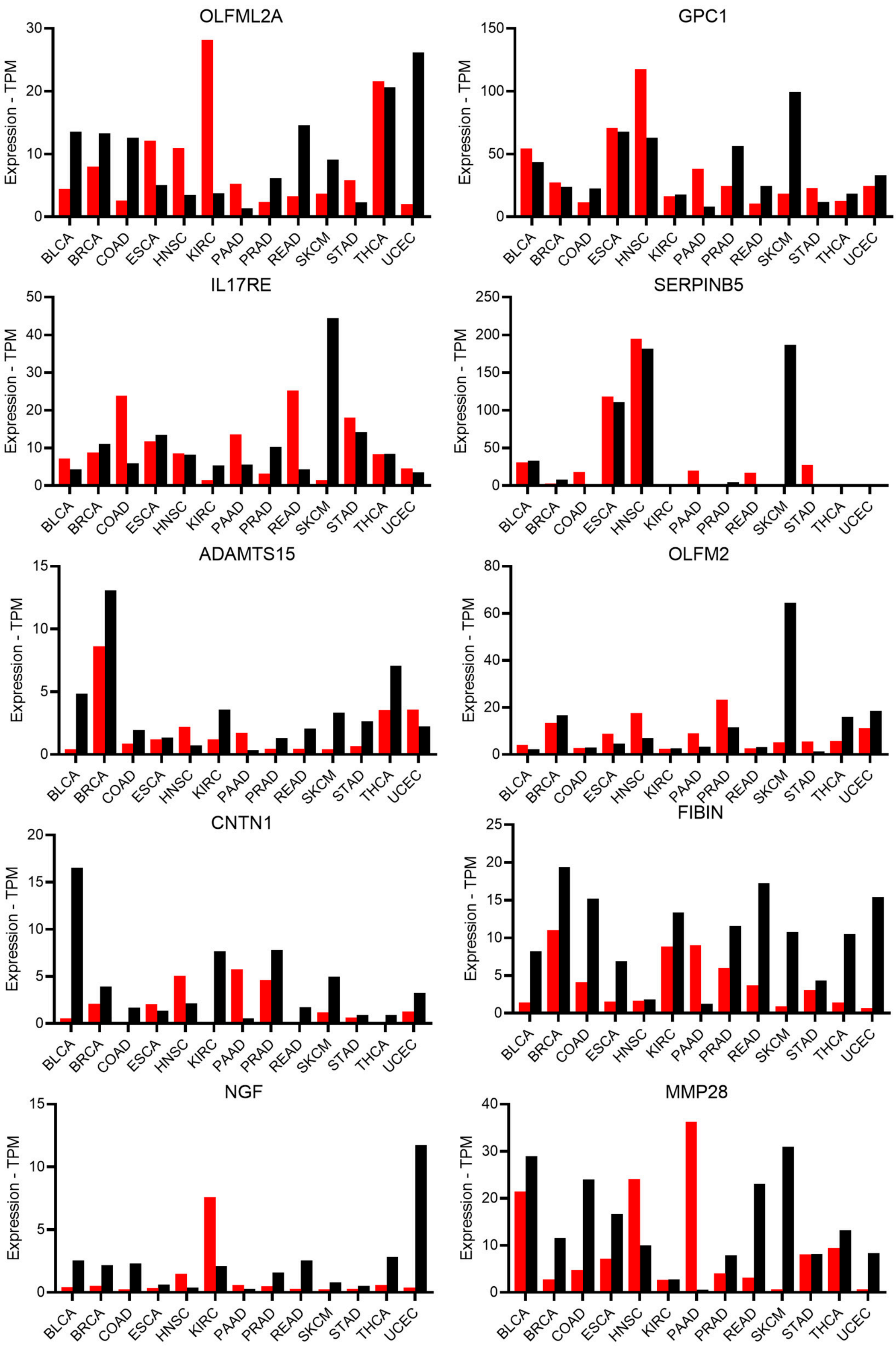

Figure S5. Transcriptomic analysis of some of the biomarker candidates highly expressed in normal tissues. Red and black boxes represent tumour and normal tissues, respectively. BLCA, bladder urothelial carcinoma; BRCA, breast invasive carcinoma; COAD, colon adenocarcinoma; ESCA, Esophageal carcinoma; KIRC, kidney renal clear cell carcinoma; HNSC, head and neck squamous cell carcinoma; PAAD, pancreatic adenocarcinoma; PRAD, prostate adenocarcinoma; READ, rectum adenocarcinoma; SKCM, skin cutaneous melanoma; STAD, stomach adenocarcinoma; THCA, thyroid carcinoma; UCEC, Uterine Corpus Endometrial Carcinoma.

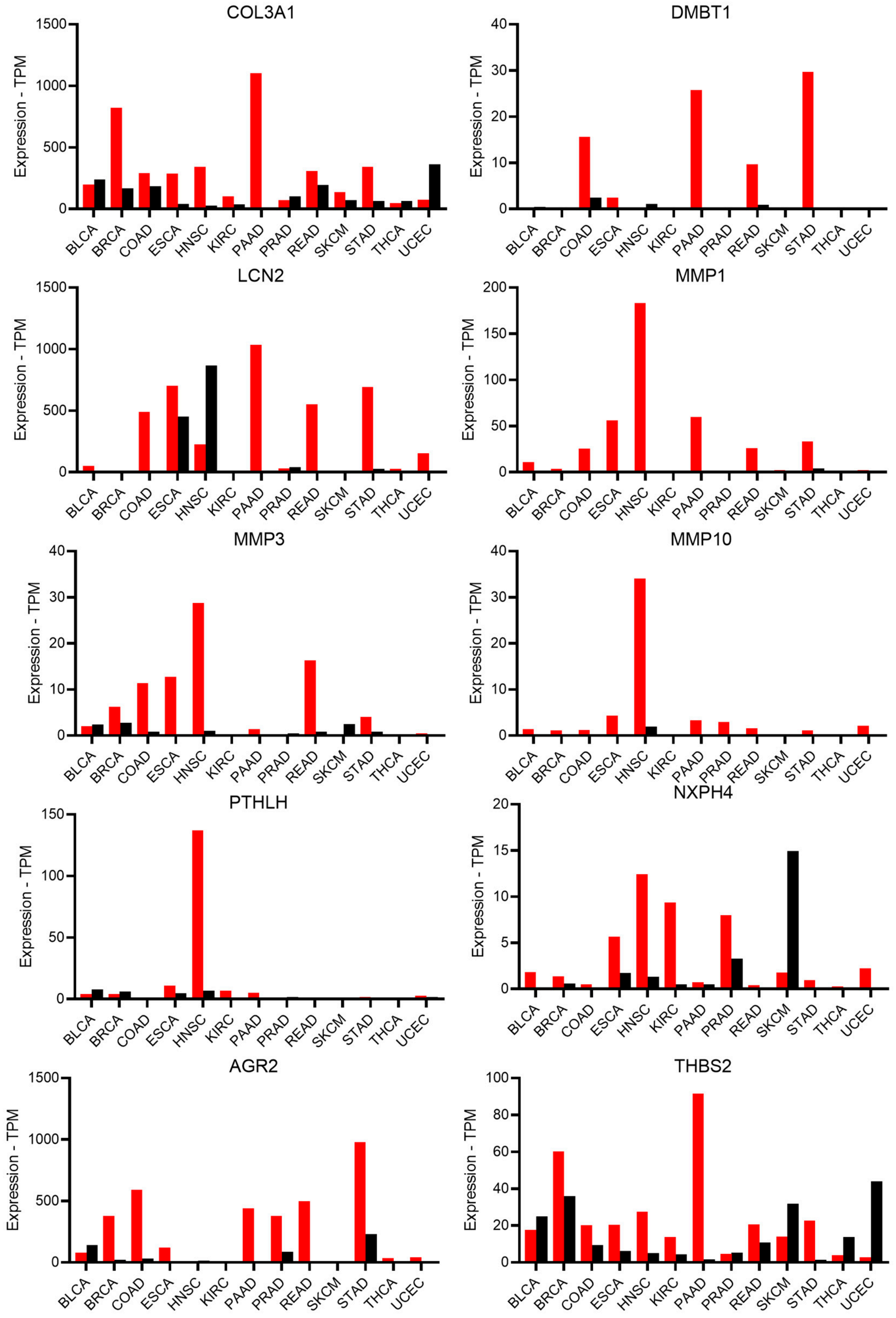

Figure S6. Transcriptomic analysis of biomarker candidates highly expressed in tumor tissues compared with normal tissues. Red and black boxes represent tumour and normal tissues, respectively.

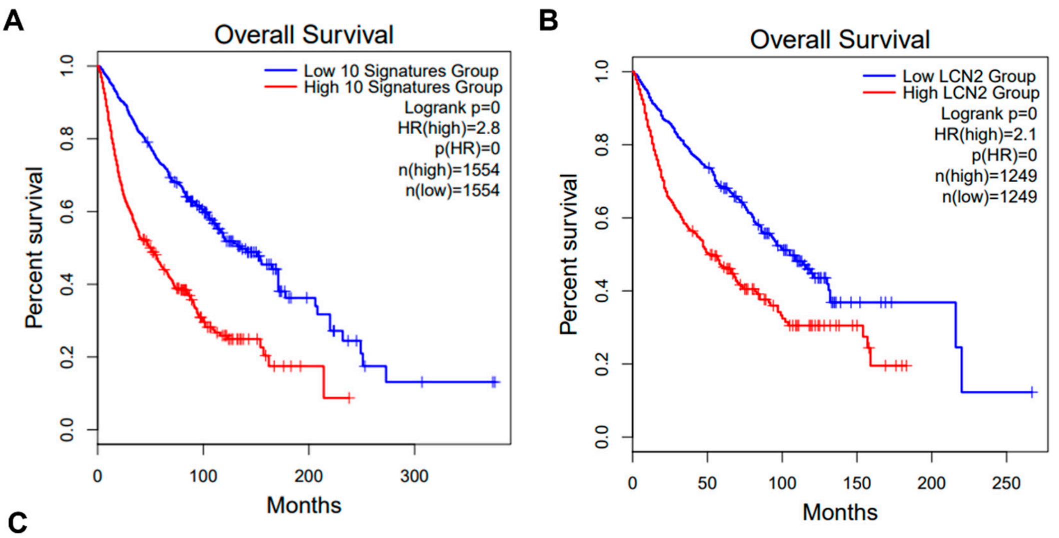

C

	BLCA	BRCA	CESC	ESCA	HNSC	KIRC	LIHC	LUSC	PAAD	READ	STAD	THCA
COL3A1	1.57							1.43			1.68	
DMBT1			1.59	1.95	1.5	1.53		1.36				
LCN2	1.45					2.1		1.37				
MMP1		2.31	2.18		1.39	1.62	2.15	1.49	2.22		1.55	3.35
MMP3			2.15		1.4	2.33	2.09		1.94			
NXPH4	1.62	2.04		2.36			2			2.85		
PTHLH	1.59		1.95			1.69	1.85	1.38	1.71		1.71	3.62
THBS2	1.62					1.51		1.39	1.57		1.69	
AGR2				1.93		1.89	2.02					
MMP10				2.03	1.37		2.48		1.66		1.65	

Figure S7. Correlations between levels of biomarker candidates and survival in cancer patients. **(A)** Kaplan-Meier analysis of 10 biomarker signatures and survival in overall cancer patients with different cancer types. **(B)** Kaplan-Meier analysis of LCN2 level and survival in overall cancer patients with different cancer types. **(C)** Summary of hazard ratio (HR) of each biomarker candidate on differentiating survival for each cancer type. Only HR values with $p < 0.05$ are presented.

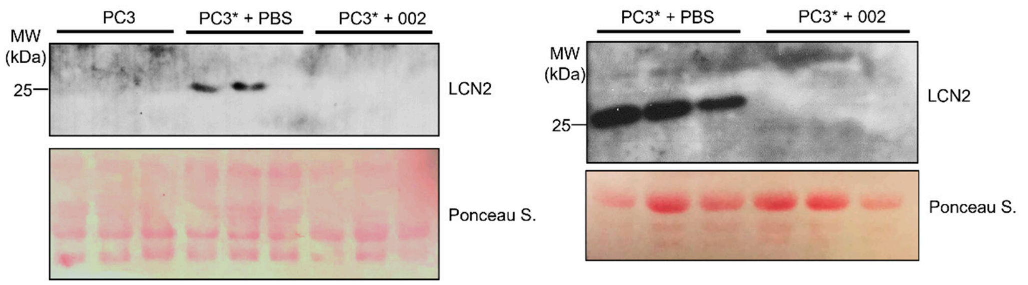

Figure S8. Western blotting of LCN2 in serum samples from PC3* model. Serum samples were collected from mice bearing PC3 or PC3* tumors and 2 μL of serum were loaded for western blot. Ponceau staining was performed to show the loading of each sample.

Table S1. ddPCR primer sequences.

Genes	Sense Primer (5′-3′)	Antisense Primer (5′-3′)
mm_Igf1	TACTTCAACAAGCCCACAGGC	ATAGAGCGGGCTGCTTTTGT
mm_Murf1	AGGTGTCAGCGAAAAGCAGT	CCTCCTTTGTCCTCTTGCTC
mm_Atrogin-1	GTTTTCAGCAGGCCAAGAAG	TTGCCAGAGAACACGCTATG
mm_Mstn	AGAAGATGGGCTGAATCCCTTT	ATCGCAGTCAAGCCCAAAGT
mm_Ucp1	AAGCTGTGCGATGTCCATGT	AAGCCACAAACCCTTTGAAAA
mm_Dio2	GTCCGCAAATGACCCCTTT	CCCACCCACTCTCTGACTTTC
mm_Cidea	GGTTCAAGGCCGTGTTAAGG	CGTCATCTGTGCAGCATAGG
mm_Pgc1a	AGACAAATGTGCTTCGAAAAAGAA	GAAGAGATAAAGTTGTTGGTTTGGC
hs_Olfm2	GGGAGGAGGTGAGGAATCTC	CCATCGTGTCAGTCATCCAG
hs_Klk5	GCAGGTAGAGACTCCTGCCA	CACAAGGGTAATCTCCCCAG
hs_Klk6	GGTGCTGAGTCTGATTGCT	CGCCATGCACCAACTTATT
hs_Thbs2	TCGTGCGCTTTGACTACATC	GTGCCGTCAATCCAGTAGGT
hs_Serpinb2	CCTGATGCGATTTTGCAGG	CGCAGACTTCTCACCAA
hs_Pnliprp3	GCCAGGCATGACTTACACAA	ACCATTTCTGCTCCCAACTT
hs_Defb1	TGTCTGAGATGGCCTCAGGT	GGGCAGGCAGAATAGAGACA
hs_Clca2	ACCCTATCTTGGACAGCACCTGGAGAA	CTTGGATATTCTGTAGACTTTTACTCA
hs_Scg5	GGGCAAGTGGAACAAGAAAC	ACATTATCCAGTCTCTGTCCTTG
hs_Mmp3	AGCAAGGACCTCGTTTTCATT	GTCAATCCCTGGAAAGTCTTCA
hs_Nectin1	ACATCTGCGAGTTTGCTACC	GTTCCGGATCTCCTGGTACT
hs_Pthlh	GCAGAAATCCACACAGCTGA	CGTCTCCACCTTGTTAGTTTCC

Table S2. Clinical characteristics of cohorts in clinical trial NCT04127981.

Variables	Non-cachectic (*n* = 6)	Cachectic (*n* = 6)	*p* value
Age (years)	52 ± 11.3	65 ± 9.1	0.0577
BMI (kg/m^2)	25.3 ± 1.9	20.0 ± 3.0	0.0041
SMI (kg/m2)	14.3 ± 1.17	14.2 ± 1.88	0.8717
Appen. SMI (kg/m2)	5.8 ± 0.41	5.6 ± 0.85	0.4833
Total fat DEXA %	42.1 ± 1.12	26.3 ± 8.34	0.0010
Total fat ldCT %	44.8 ± 2.84	21.7 ± 11.17	0.0006
Disease stage (III/IV)	3/3	1/5	

BMI, Body Mass Index; SMI, Skeletal Muscle Index; IdCT, Incremental dynamic Computed Tomography. Values are presented as mean ± SD. P values were calculated by Student's t-test.

Table S3. Detailed information for individual patients in clinical trial NCT04127981.

	Age (years)	BMI (kg/m^2)	SMI (kg/m^2)	Appen. SMI (kg/m^2)	Total fat DEXA %	Total fat ldCT %	Cancer stage	Cancer Type
NCC-1	58	26.9	6.03	14.8	43.7	46.5	IV	Colorectal
NCC-2	45	25.2	5.61	13.9	42.9	47.1	IV	Colorectal
NCC-3	70	27.5	6.44	15.8	42.6	42.7	III	Colorectal
NCC-4	53	23.8	5.29	12.9	41.1	40.5	III	Colorectal
NCC-5	51	22.5	5.62	13.2	41	44.4	IV	Colorectal
NCC-6	37	26	6.03	15.3	41.3	47.8	III	Colorectal
CC-1	56	20.3	6.22	15	21.9	22	IV	Pancreatic
CC-2	54	16.3	5.07	13.2	18.5	7.8	IV	Pancreatic
CC-3	73	18.4	4.15	10.9	39.1	30	IV	Pancreatic
CC-4	76	17.8	5.98	14.4	18.2	8.8	IV	Pancreatic
CC-5	62	23.4	6.43	16.2	28.1	26.8	III	Pancreatic
CC-6	69	23.5	5.49	15.3	32.2	34.7	IV	Pancreatic

NCC represents normal cancer patients without cachexia; CC represents patients with cachexia.
